# Coronary Venous Pressure and Microvascular Hemodynamics in Patients With Microvascular Angina: A Randomized Clinical Trial

**DOI:** 10.1001/jamacardio.2023.2566

**Published:** 2023-08-23

**Authors:** Helen Ullrich, Philipp Hammer, Maximilian Olschewski, Thomas Münzel, Javier Escaned, Tommaso Gori

**Affiliations:** 1 Department of Cardiology, Cardiology I, University Medical Center Mainz, Mainz, Germany; 2 German Centre for Cardiovascular Research (DZHK), Partnersite RheinMain, Mainz, Germany; 3 Helios Kliniken Schwerin, Schwerin, Germany; 4 Hospital Clinico San Carlos, Madrid, Spain

## Abstract

**Importance:**

The role of the coronary venous circulation in regulating myocardial perfusion and its potential in treating microvascular angina is unexplored.

**Objective:**

To evaluate whether an increase in coronary venous pressure modifies microvascular resistance in patients with microvascular angina.

**Design, Setting, and Participants:**

This was a blinded, sham-controlled, crossover, randomized clinical trial that enrolled participants between November 2021 and January 2023. Participants for this physiology end point study were recruited from the Cardiology Center of the University of Medicine in Mainz, Germany. Patients with moderate/severe angina pectoris (Canadian Cardiovascular Society class 2-4) due to microvascular dysfunction (as defined by the thermodilution-based index of microvascular resistance >25 mm Hg × s). Exclusion criteria were epicardial coronary disease, second- and third-degree atrioventricular block, severe valvular heart disease, cardiomyopathy, and pulmonary or kidney disease.

**Intervention:**

Inflation of an undersized balloon placed in the cardiac coronary sinus (CS), hereafter referred to as *balloon* and the deflated balloon in the right atrium, referred to as *sham*. Measurements were performed at rest and during maximal coronary hyperemia. Both patients and final assessors were blinded to the randomization sequence.

**Main Outcomes and Measures:**

Hemodynamic parameters, including aortic (Pa) and distal (Pd) coronary pressure, coronary sinus pressure (Pcs), right atrial pressure (Pra), and the mean transit time (inverse of blood flow [Tmn]), were measured.

**Results:**

A total of 20 patients (median [IQR] age, 69 [64-75] years; 11 female [55.0%]) were included in the study. Two patients (10%) had diabetes, 6 (30%) had hypercholesterolemia, 15 (75%) had hypertension, and 3 (15%) were active smokers. The inflation of the CS balloon caused a significant increase in CS pressure at rest and during hyperemia (300% and 317% increase, respectively, compared with sham, both *P* < .001), a decrease in hyperemic distal coronary pressure (median [IQR], sham: 92 [80-100] mm Hg; balloon: 79 [75-93] mm Hg; *P* = .01) and mean transit time (sham: 0.39 [0.23-0.62] s; balloon: 0.26 [0.17-0.46] s; *P* = .008). As a result, CS occlusion led to a decrease in both resting coronary resistance (median [IQR], sham: 59 [37-87] mm Hg × s; balloon: 42 [31-67] mm Hg × s; *P* = .005) and the primary end point hyperemic coronary resistance (mean [IQR], sham: 31 [23-53] mm Hg × s; balloon: 14 [8-26] mm Hg × s; *P* < .001).

**Conclusion and Relevance:**

Increased coronary venous pressure led to a reduction of microvascular resistances in patients with microvascular angina, a mechanism with potential implications for the therapy of this complex disease.

**Trial Registration:**

ClinicalTrials.gov Identifier: NCT05034224

## Key Points

**Question:**

How does coronary venous pressure influence myocardial resistances in patients with microvascular disease?

**Findings:**

In this blinded, physiology end point, sham-controlled randomized clinical trial that included 20 adults with microvascular disease, an increase in coronary venous pressure led to significantly decreased resting and hyperemic microvascular resistances.

**Meaning:**

In patients with microvascular angina, the coronary venous circulation may be a novel therapy target.

## Teaser

This randomized clinical trial investigates the effect of coronary venous pressure on myocardial resistances in patients with microvascular disease.

## Introduction

Microvascular angina pectoris (MVA) is a complex clinical condition in which functional and/or structural changes in the coronary microcirculation determine an increase in vascular tone compromising myocardial perfusion.^1^

Empirical observations suggest that implantation of a coronary sinus reducer may improve myocardial perfusion and decrease symptoms in patients with microvascular angina^2^ as it does in patients with refractory angina due to coronary artery disease.^3^ However, the physiologic mechanisms of this observation and the role of the coronary venous circulation in modulating (microvascular) hemodynamics remain unclear.

The study presented here aimed to investigate whether an increase in coronary sinus pressure may lead to a measurable change in coronary microvascular resistance in patients with microvascular dysfunction, which may pave the way for an interventional approach.

## Methods

### Study Design

This study was a sham-controlled, crossover, randomized clinical trial to investigate the effect of changes in coronary venous pressure on microvascular resistances. The protocol (described in detail elsewhere; Supplement 1)^4^ was approved by the University of Mainz ethics committee, and all patients provided written informed consent before enrollment. This study followed the Consolidated Standards of Reporting Trials (CONSORT) reporting guidelines.

### Study Population

Patients had chronic angina (Canadian Cardiovascular Society 2-4) without epicardial stenosis, reversible ischemia on noninvasive testing, and evidence of microvascular dysfunction (index of microvascular resistance [IMR] ≥25 mm Hg × s). Main exclusion criteria were second- or third-degree atrioventricular block, any valvular heart disease, any cardiomyopathy, and pulmonary or kidney disease.

### Procedures

#### Randomization and Intervention

**Figure 1.**
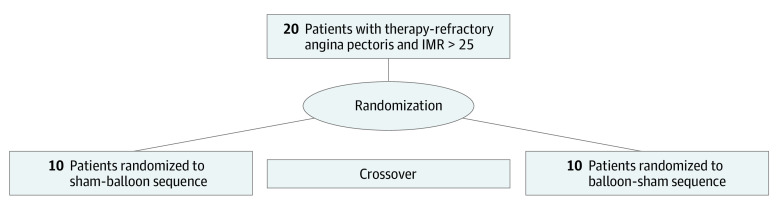
Study Protocol A total of 20 patients with microvascular angina were randomly assigned to treatment in this crossover, sham-controlled trial to assess the effect of inflation of a coronary sinus balloon on myocardial hemodynamic. IMR indicates index of microvascular resistance.

Vasoactive therapies were suspended for more than 12 hours before measurements were taken. Patients were randomly assigned 1:1 to 1 of 2 arms (sham/balloon or balloon/sham), with each patient undergoing invasive measurements at rest and during hyperemia in both conditions (Figure 1). For the measurements during balloon inflation, a 9-mm Swan-Ganz catheter was advanced into the cardiac coronary sinus (CS), and measurements were performed 1 minute after balloon inflation. The balloon was placed to obtain a stable approximately 80% CS lumen reduction throughout the measurements. For the sham measurements, the balloon was maintained in the deflated state. Ten minutes after the first set of rest/hyperemia measurements (balloon or sham), patients would cross over to the other condition (sham or balloon), and the measurements (rest/hyperemia) were repeated.

#### Hemodynamic Measurements

Coronary microvascular function assessments were performed with a wire-based thermodilution method using Pressure Wire X (Abbott Vascular) as per instructions for use; the same hyperemic agent was used for the 2 sets of measurements in each patient (sham and balloon). The mean transit time (Tmn), coronary flow reserve (CFR), and IMR were measured. The absence of drift was confirmed at the end of the measurements. Patients were blinded to the randomization sequence, and hemodynamic data were encoded in the CoroFlow software (Coroventis) and collected by blinded assessors. Coronary flow capacity was measured to identify 4 different regions of myocardial ischemia (eMethods in Supplement 2).^5,6^

### Trial End Points

The study’s primary end point was the (hyperemic) IMR during the inflation of the CS balloon as compared with sham. IMR was calculated as [(*Pd* − *Pcs*) × *Tmn*] where *Pd* is the distal intracoronary pressure, *Pcs* the pressure in the coronary sinus, and *Tmn* the inverse of flow.

### Statistical Analysis

Randomization was performed by a computer-generated random sequence with permuted blocks. The power calculation was based on previous articles investigating the effect of acute administration of fasudil and enalaprilat on IMR.^7,8^ Assuming a mean (SD) IMR of 33 (13) mm Hg × s, an effect size of approximately 0.69, and a power of 80%, 19 patients were needed for the analysis.

The primary analysis was performed as a within-participant comparison of the primary parameter using a linear mixed-regression model with a patient random intercept, condition, and period as well as their interaction as fixed factors and age and sex as covariates. Secondary end points were analyzed using the Wilcoxon test. Data are presented as median (IQR). All *P* values were 2-sided, and *P* < .05 was considered statistically significant. Data were analyzed using MedCalc software, version 15 (MedCalc Ltd).

## Results

A total of 20 patients (median [IQR] age, 69 [64-75] years; 11 female [55.0%]; 9 male [45%]) were enrolled in this physiology end point study (eTable in Supplement 2). Two patients (10%) had diabetes, 6 (30%) had hypercholesterolemia, 15 (75%) had hypertension, and 3 (15%) were active smokers. Ten patients were randomly assigned to the sham-balloon sequence and 10 to the balloon-sham sequence. The median (IQR) IMR measured before inclusion in the study was 36 (33-71) mm Hg × s (normal <25 mm Hg × s).

Hemodynamic data are presented in the Table. Inflation of the CS balloon caused a significant increase in CS pressure both at rest (300% vs sham; *P* < .001) and during hyperemia (317%; *P* < .001), but it was not associated with a change in right atrial pressure. A decrease in hyperemic distal coronary pressure was also observed (median [IQR], sham: 92 [80-100] mm Hg; balloon: 79 [75-93] mm Hg; *P* = .01) along with a decrease in Tmn (median [IQR], sham: 0.39 [0.23-0.62] s; balloon: 0.26 [0.17-0.46] seconds, *P* = .008). Balloon inflation provoked a decrease in both resting coronary resistance (median [IQR], sham: 59 [37-87] units; balloon: 42 [31-67] mm Hg × s; *P* = .005) and hyperemic coronary resistance (median [IQR] primary end point, sham: 31 [23-53] mm Hg × s; balloon: 14 [8-26] mm Hg × s; *P* < .001) (Figure 1). The estimated adjusted between-conditions difference (sham-balloon) was 21 (95% CI, 14-28 mm Hg × s; *P* < .001). Balloon inflation also caused a decrease in fractional flow reserve (median [IQR], sham: 0.94 [0.88-0.94]; balloon: 0.87 [0.82-0.94]; *P* = .003).

**Table.** Hemodynamic Variables and Effects of Balloon Inflation at Rest and During Hyperemia^a^

Hemodynamic variable	Sham	Balloon	*P* value^b^
	Median (IQR)	Median (IQR)	
Primary end point			
IMR, mm Hg × s	31 (23-53)	14 (8-26)	<.001
Secondary end points			
Rest			
Pa, mm Hg	103 (93-110)	101 (89-111)	.28
Pd, mm Hg	98 (85-101)	89 (84-102)	.21
Tmn, s	0.69 (0.43-1.14)	0.58 (0.44-0.82)	.37
Pcs, mm Hg	5 (2-9)	20 (13-29)	<.001
Pra, mm Hg	4 (2-7)	3 (2-8)	.63
Resistances, mm Hg × s	59 (37-87)	42 (31-67)	.005
Hyperemia			
Pa, mm Hg	98 (88-110)	89 (84-102)	.05
Pd, mm Hg	92 (80-100)	79 (75-93)	.01
Tmn, s	0.39 (0.23-0.62)	0.26 (0.17-0.46)	.008
Pcs, mm Hg	6 (3-9)	25 (13-36)	<.001
Pra, mm Hg	6 (3-8)	5 (3-8)	>.99
FFR	0.94 (0.88-0.94)	0.87 (0.82-0.94)	.003
CFR	1.70 (1.4-2.3)	2.1 (1.3-4.1)	.18
MRR	2.0 (1.4-2.7)	2.7 (1.4-5.3)	.06

Abbreviations: CFR, coronary flow reserve; FFR, fractional flow reserve; IMR, index of microvascular resistance; MRR, microvascular resistance reserve; Pa, mean aortic pressure; Pcs, mean pressure in the coronary sinus; Pd, mean distal coronary pressure; Pra, mean pressure in the right atrium; Tmn, mean transit time.

^a^ The estimated adjusted between-conditions difference (sham-balloon) was 21 (95% CI, 14-28) mm Hg × s (*P* < .001).

^b^ All *P* values are calculated with the Wilcoxon test.

Randomization sequence, resting resistances, sex, and age did not affect the change in IMR caused by balloon inflation. The decrease in IMR after balloon inflation was largest in patients with the highest sham IMR (*R^2^* = 0.67; *P* < .001) (eFigure 2 in Supplement 2).

**Figure 2.**
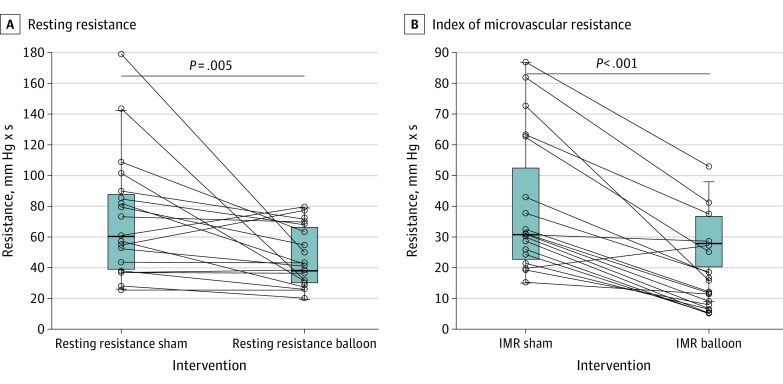
The Primary End Point of the Trial Balloon inflation caused a decrease in resting and hyperemic microvascular resistances. IMR indicates index of microvascular resistance.

Coronary flow capacity was improved by balloon inflation (Figure 2 and eFigure 1 in Supplement 2). Average coronary flow capacity (CFC) went from moderate myocardial ischemia (sham: CFC = 1.7; 1/Tmn = 2.56 s) to the nonischemic region (balloon: CFR = 2.1; 1/Tmn = 3.89 s).

## Discussion

Many patients undergoing diagnostic coronary angiography for angina have microvascular disease.^8^ These patients rarely receive a definitive diagnosis in clinical routine and often remain symptomatic.^9,10^ To date, there is little evidence of the possibility of modulating microvascular resistances using pharmacologic or mechanical approaches. Small case series support the concept that occlusion of the CS may improve symptoms in these patients, but the mechanism is unknown.^2^ We show here that an acute increase in CS pressure leads to a decrease in microvascular resistance (with a more significant effect during hyperemia) and an increase in blood flow.

Animal models in the peripheral circulation show that total vascular resistance within the coronary circulation is determined by vascular resistance and viscosity, with the latter playing a multiplicative role during hyperemia when the capillary bed determines up to 75% of the total myocardial vascular resistance.^11^ In the healthy circulation, increases in venous blood pressure elicit myogenic vasoconstrictor response of precapillary vessels and capillary derecruitment.^12^ In patients with severe vascular disorders, however, an increase in venous pressure has been associated with a rise in capillary (transmural) pressure, leading to an increase in capillary diameter and density (and therefore total blood volume) and a reduction in viscosity.^13^ Based on the Poiseuille law, volume flow rate is directly proportional to the fourth power of the vessel’s radius and inversely proportional to dynamic viscosity. Following an increase in CS pressure, even small increases in capillary recruitment/diameter and viscosity may, therefore, result in relevant improvements in microvascular resistance in patients with MVA, with a proportionally larger effect during hyperemia (eFigure 3 in Supplement 2).

### Limitations

This study has some limitations. Of note, a mechanistic study is not designed or powered to assess clinical benefit, and we only studied the effect of a single acute increase in CS pressure in patients with microvascular dysfunction. Whether similar effects occur over chronic periods and in patients with epicardial (as compared with microvascular) disease will have to be investigated. Also, the mechanisms hypothesized are based on evidence from animal models or human in vivo models of the extracardiac circulation.^11,12,13^

## Conclusions

In this crossover randomized clinical trial, the current findings that an acute increase in CS pressure led to a decrease in microvascular resistance and an increase in blood flow allow hypothesizing that the venous vasculature may be leveraged to improve coronary resistances in patients with MVA. This may have implications for therapy with a coronary sinus reducer.

## Notes

Supplement 1. Trial Protocol

Supplement 2. **eTable.** Patient Characteristics
eMethods.
**eFigure 1.** Impact of Balloon Expansion on Coronary Flow Capacity **eFigure 2.** Correlation Between the Decrease in IMR Following Balloon Inflation and Sham (Resting) IMR **eFigure 3.** Proposed Mechanism
eReferences.

Supplement 3. Data Sharing Statement
