# Supplementary material for: Coronary Venous Pressure and Microvascular Hemodynamics in Patients With Microvascular Angina: A Randomized Clinical Trial
Source: JAMA Cardiol. 2023 Aug 23;8(10):979–83. doi: 10.1001/jamacardio.2023.2566 (PMC10448373; doi:10.1001/jamacardio.2023.2566)
Supplement: Supplement 1. — Trial Protocol [file jamacardiol-e232566-s001.pdf]

Clinical Investigation Plan

Study Title: MACCUS: ModulAtion of Cardiac Coronary sinus hemodynamics to develop a new treatment for microvascUlar diseaSe. A single center, randomized study.

Protocol acronym	MACCUS
Clinical Study Reference Number	NCT05034224
Version	1.0
Date	16/07/2020
Sponsor	*Universitätsmedizin Mainz*<br>*Zentrum für Kardiologie, Kardiologie I*<br>*Direktor: Univ-Prof. Dr. med. Thomas Münzel*<br>*Langenbeckstraße 1*<br>*55131 Mainz*<br>*Tel. (06131) 17 2829*<br>*Fax (06131) 17 6615*
Principal investigator:	Univ-Prof. Tommaso Gori, PhD<br>Universitätsmedizin Mainz

CLINICAL INVESTIGATION PLAN HISTORY

Version	Date	Main changes
1.0	16/07/2020	First submission

Responsibilities

**Coordinating Clinical Investigators / „Leiter der Klinischen Prüfung“ in Germany**	Univ-Prof. Tommaso Gori, Dott Med e Chir (Siena), PhD (Toronto)
**Sponsor**	Universitätsmedizin Mainz, Zentrum für Kardiologie, Kardiologie I, Direktor: Univ-Prof. Dr. med. Thomas Münzel, Langenbeckstraße 1, 55131 Mainz, Tel. (06131) 17 2729, Fax (06131) 17 6615
**CRO**	Not applicable
**Start up activities**	Not applicable
**Steering Committee**	Not applicable
**Trial Statistician**	Christian Ruckes, IZKS Universitätsmedizin Mainz
**Responsible Data Manager**	Univ-Prof. Tommaso Gori, Dott Med e Chir (Siena), PhD (Toronto)
**Responsible for Vigilance and Regulatory Support**	Not applicable
**Other members of the study group**	Maximilian Olschewski<br><br>Dr. Helen Ullrich

Signatures

Compliance Statement

The studies will be conducted in accordance with the design and specific provisions of this CIP, in accordance with the ethical principles that have their origin in the Declaration of Helsinki, and that are consistent with Good Clinical Practice (GCP) and the applicable regulatory requirements.

We, the undersigned, have read and approve the CIP specified above and agree on its content.

**Coordinating Clinical Investigators / „Leiter der Klinischen Prüfung" in Germany**	*Prof. Tommaso Gori, Dott Med e Chir (Siena), PhD (Toronto)*<br>13.09.2020<br>Signature Date
**Sponsor**<br>**Director of the clinic**	*Prof. Dr. med. Thomas Münzel*<br>13.09.2020<br>Signature Date<br>Universitätsmedizin Mainz, Zentrum für Kardiologie, Kardiologie I<br>Direktor: Univ-Prof. Dr. med. Thomas Münzel

Signature of the Principal Clinical Investigator

Name	Tommaso - Gori, MD, PhD
Institution	Zentrum für Kardiologie, Kardiologie I
Street	Langenbeckstr. 1
ZIP Code / City	55131 Mainz
Country	DE

I have read this protocol, appendices and amendment(s), if applicable, and agree to adhere to the requirements. I will provide copies of this protocol and all pertinent information to the study personnel under my supervision. I will discuss this material with them and ensure they are fully informed regarding the device and the conduct of the studies.

I will conduct the studies in accordance with the protocol, the Declaration of Helsinki, Good Clinical Practice guidelines as well as local regulations, and I accept respective revisions to the protocol approved by authorized personnel of the Sponsor and by regulatory authorities.

I agree to report every occurring Adverse Event and Device Deficiency according to the timelines and regulations indicated in the CIP.

Mainz, 25th August 2020	
City, Date	Signature of Principal Investigator

1 Table of Contents

Responsibilities 3

Signatures 3

1 Table of Contents 6

2 List of Abbreviations 8

3 Synopsis 10

4 Background and Study Rationale .... **Error! Bookmark not defined.**

PROTOCOL RATIONALE 12

BACKGROUND 12

5 Hypothesis 14

6 Study design 14

7 Trial Endpoints 14

Primary endpoint 14

Secondary endpoints: 14

8 Procedures 15

9 Study population 17

Inclusion criteria 17

Patients unable to understand the scope of the study/unable to consent 17

10 Other procedures 18

Recruitment 18

Pre-interventional Procedures 18

Randomization 18

Post-interventional procedures 19

11 Risks and benefits 20

Risks *20*

Benefits: *20*

12 Study insurance 20

13 Information sharing with the practitioner 21

14 Data management 21

15 Monitoring 21

16 Use of radiation 21

17 Statistical analysis 21

Power Calculation *21*

Statistical analysis of primary and secondary endpoints *22*

18 References 22

19 Financing 23

20 Publication Policy 23

109 2 List of Abbreviations

**ACS**	Acute Coronary Syndrome
**AE**	Adverse Event
**BMI**	Body-Mass-Index
**CABG**	Coronary Artery Bypass Graft
**CAD**	Coronary artery disease
**CCI**	Coordinating Clinical Investigator / Leiter der Klinischen Prüfung
**CCS**	Canadian cardiovascular society
**CE**	CE mark, a stylized "CE" (Conformité Européenne) placed on products to signify conformance with European Union regulations
**CFR**	Coronary flow reserve
**CIP**	Clinical Investigation Plan
**CRO**	Clinical Research Organization
**EC/MEC**	Ethics Committee/Medical Ethics Committee
**ECG**	Electrocardiogram
**FFR**	Fractional flow reserve
**GCP**	Good Clinical Practice
**FFR**	Fractioned flow reserve
**hrs**	Hours
**IMR**	Index of microvascular resistances
**INR**	International normalized ratio
**ISO**	International Organization for Standardization
**LBBB**	Left Bundle Branch Block
**Pa**	Pressure, aortic
**Pd**	Pressure, distal
**PB-CFR**	Pressure-bound CFR
**PCI**	Percutaneous Coronary Intervention
**aPTT**	Activated thromboplastin time
**RFR**	Resting full-cycle flow ratio
**SAE**	Serious Adverse Event
**SC**	Steering Committee
**SD**	Standard deviation
**Tmn**	Mean transit time

URL upper reference limit

3 Synopsis

Title	**M**odul**A**tion of **C**ardiac **C**oronary sinus hemodynamics to develop a new treatment for microvasc**U**lar disea**S**e**.**
Acronym	MACCUS
Type of study	Randomized, single-center, controlled, cross-over interventional study. Coronary artery resistances will be measured at baseline and during temporary occlusion of the coronary sinus. All patients will have an indication for assessment of microvascular function as a prerequisite (and independently of) their participation in the study.
Medical condition	Coronary microvascular disease, refractory angina
Study population	Patients undergoing hemodynamic assessment of microvascular function during the rule-out of myocardial ischemia / angina.
Objective of the study	To determine the acute effect of an increase in coronary sinus pressure on microvascular resistances in patients with microvascular angina;
Key inclusion criteria	Chronic coronary syndrome (including patients with anginal equivalents). Reversible ischemia on non-invasive testing, indication to cardiac catheterization; Indication to the assessment of microvascular function (note: patients will be asked for participation and consented prior to the first measurement of microvascular function, which will be conducted as per clinical indication; only those with evidence of pure or mixed microvascular disease (index of microvascular resistances (IMR)>25 will proceed into the study); Willingness to participate and ability to understand, read and sign the informed consent; age>18 years
Key exclusion criteria	Previous CABG with patent grafts to the left anterior descending coronary<br>Epicardial coronary disease (FFR <0.80 with evidence of a focal stenosis) in the left anterior descending territory<br>Severe valvular heart disease<br>Any cardiomyopathy; pulmonary or renal disease<br>Inability to provide informed consent<br>Any disease reducing life expectancy
Endpoints	Primary efficacy endpoint:<br>The primary endpoint is the Index of coronary microvascular resistances (IMR) in sham versus balloon condition.<br><br>Key secondary endpoint(s):<br>Physiological parameters (Pd/Pa, Tmn, CFR, RFR,

	RRR, see definitions below)<br><br>Assessment of safety:<br>Evidence of coronary ischemia, bleeding, stroke, embolization, perforation.
Risks and benefits<br>(without an expected difference between groups)	The hemodynamic measurements and accompanying examinations are on-label and applied according to clinical indication. The angioplasty balloon is expanded incompletely and at low pressure, so there is no relevant risk of vessel injury. Vascular access is performed using the femoral vein, the risks correspond to those of a routinely performed venous puncture.
Study Sites	Mainz
Patient Enrolment	up to 25
Patient Follow-Up	None
Concomitant Therapy	As per guidelines
Timelines<br>(planned)	First patient in: 09-2020<br>Last patient out. 06-2022
Statistical Analysis	Efficacy/Test accuracy:<br>The index of microvascular resistance is a well validated, gold-standard method to assess the coronary microcirculation.<br>Description of the primary efficacy/Test accuracy analysis and population:<br>The primary analysis will be performed as within-subject-comparison of the primary outcome between each of the two groups (sham or balloon).<br>Effect size assumed for estimation of sample size:<br>We expect a difference of the balloon versus sham treatment of 9 units and a within-subjects standard deviation of 13 units. Therefore, the expected effect size is 0.69.<br>Safety:<br>The safety and efficacy of all devices has been previously shown in large clinical trials. The hemodynamic measurements and accompanying examinations are on-label and are used according to the clinical indication. There are no additional risks from participation to the study.<br>Secondary endpoints (at rest and during hyperemia):<br>FFR: fractional flow reserve (a gold standard for the assessment of epicardial coronaries)<br>CRF: coronary flow reserve (a mixed index of macro- and microvascular hemodynamics)<br>IMR: index of microvascular resistance (a gold standard for the assessment of coronary microcirculation)<br>RRR: Resistance Reserve Ratio (a marker of microvascular reactivity)

	CFR_norm: CFR normalized by FFR<br>CFI: collateral flow index<br>PB-CFR: Pressure bounded CFR: CFR estimated from resting & hyperemic pressure gradients<br>Pd, Pa: peripheral and aortic pressure<br>Tmn: mean transit time
Sample Size	To be assessed for eligibility (n = 300)<br>To be allocated to trial (up to n =25)<br>To be analysed (up to n =25)

PROTOCOL SUMMARY

The aim of this study is to test whether an increase in coronary sinus pressure leads to a change in coronary microvascular resistances in patients with angina pectoris and with an indication to measurement of microvascular function as per clinical guidelines.

All patients with a clinical indication for the assessment of microvascular function will be invited to participate and will sign the informed consent at least 24 hours before the study. Patients with an index of microvascular resistances >25 (i.e. evidence of microvascular dysfunction) will undergo study-specific procedures in the same session (i.e. no additional invasive procedure is required for the study).

Study-specific procedures include the repetition of the assessment of microvascular resistances without (sham condition) versus with coronary sinus occlusion (balloon). Coronary sinus occlusion will be achieved by inflating a balloon sized to ~70% of the diameter of the coronary sinus. Since the implantation of a coronary sinus reducer is a therapeutic option for this type of patients, also this procedure (inflation of an undersized balloon in the coronary sinus) provides clinically relevant information (sizing of the vessel, effect on microvascular resistances and feasibility of the intervention).

The primary goal of the study is:

- To study the effect of coronary sinus occlusion on microvascular resistances at rest and during hyperemia.

4 Background and Study Rationale

BACKGROUND

Microvascular angina still represents a major clinical challenge whose importance is acknowledged in the most recent chronic coronary syndromes guidelines[1]. In particular, there is a strong disagreement on its classification, on the several mechanisms behind its pathophysiology [2], and the therapeutic alternatives available remain unsatisfactory.

Despite these uncertainties, there is a consensus that this condition is clinically relevant as it affects up to two-thirds of patients who suffer from stable angina and either have no epicardial coronary stenosis at angiography or have combined epicardial and microvascular disease [3].

Microvascular angina caused by a dysfunctional microvascular bed cannot be treated by standard revascularization therapies and the pharmacological strategies (e.g. nitrates, ca-channel blockers, ranolazine) available also provide limited benefit. As a consequence, the old hypothesis of improving myocardial perfusion by diverting blood from the coronary venous system to an ischemic region ("venous retroperfusion") has again gained attention during recent years. This therapy was proposed as early as 1953 and is based on the concept that an elevation in backward pressure in the coronary venous system provokes dilatation of the subendocardial arterioles, resulting in a relative reduction of vascular resistance in this area and a redistribution of blood flow to these ischemic subendocardial layers[4]. Numerous studies confirm the efficacy of this approach for the therapy of patients who have angina and are not candidates for "traditional" revascularization[4].

The study presented here aims to investigate the effect of an increase in coronary sinus pressure on microvascular function in the acute setting, a potentially new therapeutic concept.

Initial evidence from animal studies and a series of patients appears to support the hypothesis that coronary sinus occlusion (CSO) improves microvascular function: Ido et al. [10] demonstrated that coronary sinus occlusion lead to dilatation of the subendocardial arterioles, resulting in a significant reduction of vascular resistance in this area and a redistribution of blood flow to these ischemic subendocardial layers. In a small case series by Giannini et al. [11], patients with microvascular angina showed a clinical improvement after coronary sinus occlusion, an effect similar to that shown in the randomized, sham-controlled trial COSIRA [12](improvement of 2 CCS angina classes in 35% of the patients). In the paper by de Maria et al [13], percutaneous-intermittent CSO (PICSO) improved microvascular perfusion and resistances in patients with no reflow after ST-elevation myocardial infarction. Finally, we recently provided mechanistic evidence that CSO might improve microvascular function in a patient with evidence of severe microvascular dysfunction: in a recent publication (Gori T Eurointervention online), we described a case of a patient with microvascular angina that benefited from the implantation of the Reducer. This 61-year old patient with a history of insulin-dependent diabetes, hypertension, and multiple PCIs underwent implantation of a coronary sinus Reducer for chronic angina refractory to maximal therapy. His symptoms included a CCS III angina leading to 4 hospitalizations in the 6 months before Reducer implantation. In order to better understand the pathophysiology of his symptoms, a full hemodynamic assessment was performed. The fractional flow reserve (FFR) in the left anterior descending (LAD) was 0.87, witnessing the absence of epicardial disease. In contrast, the IMR was 63, documenting increased microvascular resistances as a mechanism of his symptoms. Based on this evidence, and on compassionate grounds, a coronary sinus Reducer was implanted. After implantation, the "trans-sinus" gradient was 3mmHg. FFR and resting full-cycle ratio (RFR) were unchanged (0.93 and 0.85), but IMR dropped to 37. Since implantation (currently 12 months follow-up at the time of this writing), the patient has never been admitted again to hospital and is symptom-free.

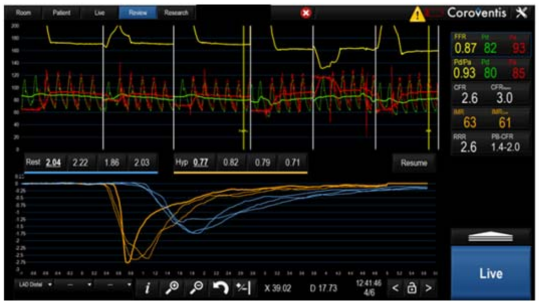

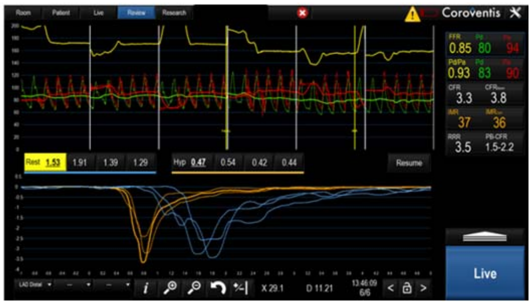

Although this is just preliminary data, they suggest that the redistribution of blood from the subepicardial to the subendocardial space might be associated with a drop in total microvascular resistances and therefore relief of symptoms.

5 Hypothesis

The hypothesis of the study is that occlusion of the coronary sinus will be associated with a decrease in the index of microvascular resistances.

6 Study design

The study is a single-center, cross-over sham controlled randomized trial to investigate the effect of coronary sinus occlusion on microvascular resistances.

7 Trial Endpoints

The primary analysis will be on the per-protocol principle (i.e. including all patients who are not protocol violators). A separate analysis will be performed on an intention-to-treat basis (i.e. all randomized patients randomized to a treatment arm).

Primary endpoint

1. Index of coronary microvascular resistances (IMR) in sham versus balloon condition.

Key Secondary endpoints:

changes in physiological parameters

1. RFR (resting flow ratio);
2. FFR (fractional flow reserve);
3. Tmn (mean transit time);
4. Pa and Pd at rest and during hyperemia (aortic and coronary pressure);
5. Hyperemic Tmn;
6. RRR: Resistance Reserve Ratio (a marker of microvascular reactivity);

7. CFR: coronary flow reserve;
8. CFR_norm: CFR normalized by FFR;
9. PB-CFR: Pressure bounded;

Note: all these parameters are assessed during an FFR/IMR measurement as clinically indicated in this patient population and do not require additional procedures or imply additional risks.

Assessment of safety:

Evidence of coronary ischemia, bleeding, stroke, embolization, perforation.

8 Procedures

Hemodynamic measurements

Coronary angiography and pressure wire assessments of coronary stenoses will be conducted as per clinical indication using standard methods. This assessment is commonly performed in the diagnostic algorithm of patients with chronic coronary syndromes without coronary stenoses. In this study, only patients in whom these assessments are planned will be included, such that all methods are used as clinically indicated as part of the normal routine.

As per standard procedures, intracoronary nitrates will be administered before each measurement when blood pressure is above 100mmHg systolic. Pressure wire normalization will be performed at the coronary ostia before each measurement and the presence of drift will be checked at each measurement. The distal position (constant across measurements) of the pressure wire will be documented fluoroscopically.

Study Procedures

Patients enrolled in the study will undergo these procedures as clinically indicated:

- Microvascular assessment will be performed in the left anterior descending coronary artery using the wire-based thermodilution method. This assessment of coronary hemodynamics will be performed as clinically indicated, according to guideline recommendations.

- Patients with FFR<0.80 and a focal stenosis (i.e. patients who can be treated with stent implantation) and/or patients with IMR<25 (i.e. without microvascular dysfunction) will be excluded from the study
- A 7-9mm balloon (sized to 70% of the coronary sinus) will be advanced in the coronary sinus, with access through the femoral vein. Microvascular function measurements will be made twice (in a random, cross-over design) during incomplete balloon occlusion ("balloon") and with the deflated balloon in the right atrium ("sham"). The inflation of the balloon is a study-specific procedure. However, this procedure provides the following information that is important for the treatment of the patient:

1. Whether the IMR drops during coronary sinus occlusion. If this is the case (as in the patient described above), implantation of a coronary sinus reducer is indicated as it would be expected to result in the reduction of angina symptoms. If the inflation of the

balloon does not lead to a drop in IMR, then a coronary sinus reducer should not be implanted.

2. In case implantation of a coronary sinus reducer is planned, the balloon allows sizing of the coronary sinus. This increases the safety of the implantation, since the most common complication during sinus reducer implantation is undersizing (resulting in device embolism) or oversizing (resulting in sinus venosus rupture).

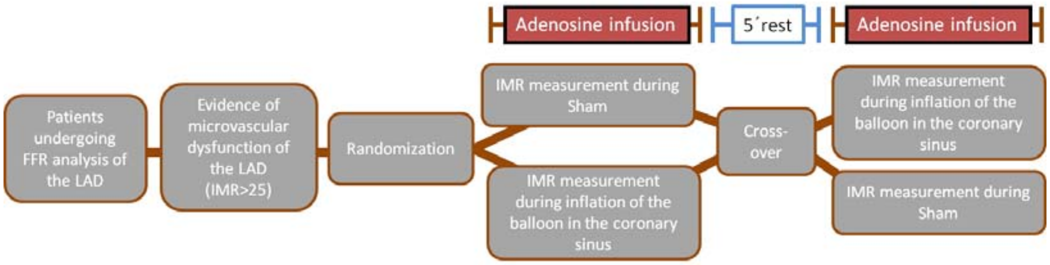

Hemodynamic recordings

Pressure wire recordings will be performed using the 0.014-inch pressure wire (Pressure Wire X, Abbott Vascular, St Clara, USA). Analysis will be performed offline using the Coroflow software (Coroventis Research AB Uppsala, Sweden).

For FFR measurements, the pressure wire will be advanced distal into the left anterior descending coronary artery (LAD). The pressure gradient between catheter (Pa, aortic pressure) and distal (wire-measured) pressure (Pd, pressure distal) will be assessed at rest and during adenosine-induced hyperemia (140microgr/kg/min i.v., papaverine 14mg i.c. in case of asthma. If the hyperemic Pd/Pa ratio (FFR, fractional flow reserve) is <0.80, a pull-back recording will be performed to look for focal stenoses.

During hyperemia, three boli of saline will be injected through the guiding catheter to assess parameters of microvascular function. All files will be coded and extracted for study purposes by blinded staff at the end of the study.

9 Safety:

Risk associated with participation in the study

All measurements (including hemodynamic measurements) are applied as clinically indicated and according to CE certificate and the instruction for use. The intervention applied in the study is limited to the repetition of the measurement of coronary physiology and the expansion of the angioplasty balloon as described above. Vascular access is performed using the femoral vein; the risks correspond to those of a routinely performed venous puncture. The measurement might have clinical relevance, since “responders” would be expected to profit more from the sinus reducer. Measurements do not require X-Rays per se. The expansion of the balloon is incomplete (only 70% of the vessel diameter) and performed using low pressure (2-4ATM). The risk of trauma is thereby minimal.

Therapies will be based on evidence-based guideline recommendation. Thus, it is not to be expected that the participation in the study will lead to unneeded or harmful therapeutic interventions.

9 Study population

The study will be conducted in patients with chronic coronary syndrome (including patients with anginal equivalents) and reversible ischemia on non-invasive testing. In the case of therapy-refractory symptoms, the indication for the implantation of a coronary sinus reducer was already clinically established in advance. There must be an indication for cardiac revascularization and an evidence of pure or mixed microvascular disease (index of microvascular resistances (IMR>25). FFR and the further haemodynamic measurements are performed routinely in our laboratory.

Inclusion criteria

Patients must meet ALL of the inclusion criteria:

Chronic coronary syndrome (including patients with anginal equivalents);
Reversible ischemia on non-invasive testing, indication to cardiac catheterization;
Indication to the assessment of microvascular function (note: patients will be asked for participation and consented prior to the first measurement of microvascular function, which will be conducted as per clinical indication; only those with evidence of pure or mixed microvascular disease (index of microvascular resistances IMR>25 will proceed into the study);
Willingness to participate and ability to understand, read and sign the informed consent;
Age>18 years

Exclusion criteria
Patients will be excluded from eligibility for study enrollment if ANY of the following criteria applies to the patient:

Previous CABG with patent grafts to the left anterior descending coronary artery
Epicardial coronary disease (FFR <0.80 with evidence of a focal stenosis) in the left anterior descending territory
Second and third degree atrioventricular block
Severe valvular heart disease
Any cardiomyopathy; pulmonary or renal disease
Inability to provide informed consent
Any disease reducing life expectancy

Patients unable to understand the scope of the study/unable to consent

Patients unable to understand the scope of the study are classified as not able to give informed consent and are excluded for eligibility for study enrollment.

Likewise, patients unable to consent, e.g. for a neurological damage, are treated as not being able to give informed consent and are excluded for eligibility for study enrollment.

10 Other procedures

Recruitment

Patients will be enrolled among those treated at Zentrum für Kardiologie, Kardiologie 1 of the Universitätsmedizin Mainz.

Patients

The decision to perform the hemodynamic assessment of the lesion will be taken before, and independently of, the participation of the patient in the study.

Consent will be obtained by study physicians before beginning of the procedure.

It is not planned to recruit patients who are not already in medical treatment in the center of cardiology of the university hospital Mainz and, therefore, it is not planned to create any recruiting material, e.g. for promotion of the study.

Pre-interventional Procedures

Per standard clinical practice, the following baseline procedures will be performed prior to enrolment:

- Medical history and physical examination, including (among others) pulses, blood pressure, height and weight (body mass index; BMI).
- 12-lead electrocardiogram (ECG).
- Screening laboratory tests: white blood cell count, hemoglobin, hematocrit, platelet count, serum creatinine, electrolytes, activated partial thromboplastin time (aPTT), international normalized ratio (INR)
- A pregnancy test will be performed in females of childbearing potential.
- transthoracic echocardiography

Randomization

Randomization will be performed after informed consent and if all inclusion criteria and no exclusion criteria are met.

Patients will be randomized 1:1 to one of the two study arms.

Randomization will be done by using a computer-generated random sequence (Medcalc, Mariakerke, BE).

Flow-chart

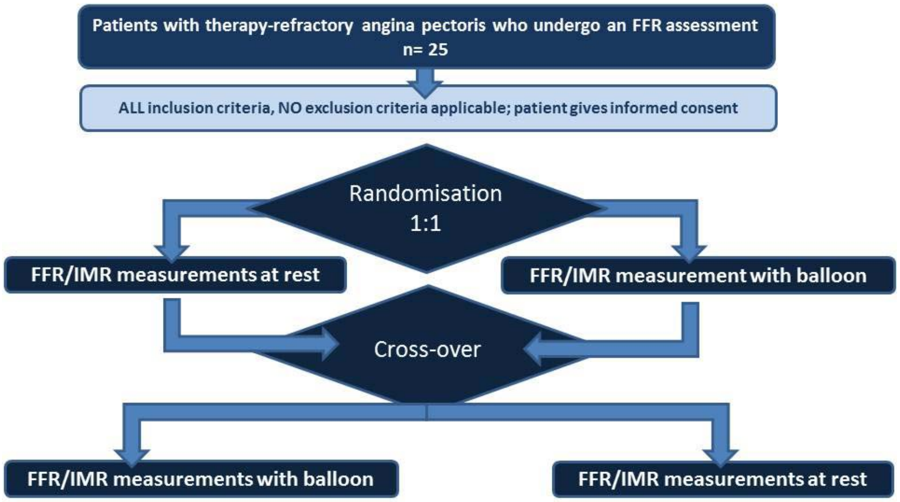

Important note:

The inflation of the balloon in the coronary sinus is a study-specific procedure, but it provides information (feasibility of coronary sinus cannulation/sizing/effect of coronary sinus occlusion on microvascular resistances) which is important for the treatment of the patient (i.e. whether implantation of a sinus reducer is indicated). All procedures are conducted based on the clinical indication, using CE-marked devices, and according to universally accepted standards.

Post-interventional procedures

After intervention, patients will be treated according to guidelines by the responsible doctor as per standard practice.

The medication treatment after intervention will be selected according to current guidelines and depending on indication of coronary intervention and severity of disease. Until discharge, patients will be treated according to routine practice, incorporating patients´ individual comorbidities.

In case additional an (staged) revascularization is deemed necessary at the time of randomization, it can be performed during index hospitalization or later and patients should be treated according to current guidelines.

11 Risks and benefits

Risks are linked with any intracoronary procedures and it is not expected that participation in the study will affect them. Access to the coronary sinus is performed through the femoral vein, which will be punctured as part of the study. The risks are similar to those of venous vessel puncture and are considered to be relatively low. The angioplasty balloon is expanded incompletely and at low pressure, so there is no relevant risk of vessel injury.

Risks

Risks linked with any intracoronary procedure:

Death, aneurysm, pseudoaneurysm, or arteriovenous fistula (AVF), stent deformation, collapse, or fracture, Emergency surgery: peripheral vascular or coronary bypass, stroke/transient ischemic attack (TIA), cardiac tamponade, coronary artery occlusion, perforation, rupture, or dissection, pericarditis, embolism (air, tissue, device, or thrombus), thrombosis (acute, subacute, late, or very late), incomplete stent apposition, myocardial infarction (MI), restenosis of the stented artery, arrhythmias, hemorrhage requiring transfusion, shock/pulmonary edema, coronary artery spasm, abrupt vessel closure, hypotension/hypertension, allergic reaction (to contrast, antiplatelet therapy, stent system—material, drug, or polymer coating), peripheral ischemia/peripheral nerve injury, infection or fever, unstable angina, access site pain, hematoma, or haemorrhage, balloon rupture, stent migration, failure to deliver the stent, stent misplacement.

Risks linked with venous catheterization:

Bleeding, hematomas, aneurysms, pseudoaneurysms, fistulas, perforations, thromboses, embolisms (air, tissue, device, or thrombus), skin/tissue and nerve damage, pain, discomfort, infections, allergic reactions

Risks linked with radiation:

Skin reaction

Benefits:

There is no benefit for the individual patients participating in the study.

12 Study insurance

Since all procedures (except for randomization) are clinically indicated and acknowledged in the current literature, a study insurance is not planned.

13 Information sharing with the practitioner

Since all procedures belong to clinical standards, is not planned to share information regarding the (non-existent) additional risks/implications connect to the participation in the study with the practitioner. A study ID will not be given out after enrollment into the study.

14 Data management

Patient data will be pseudonymized and collected by the study team.

Pseudonymized patient data will be stored digitally on an external hard-drive not connected to the clinical intranet and only accessible to the members of the study team. After ten years of storage, data will be destroyed.

It is not intended to give study participants´ data to a third party.

All data will be analysed after the last patient is discharged from index hospitalization. No interim analysis is intended. However, a Data Safety Monitoring Board consisting of two physicians not affiliated with the study will monitor the safety of the subjects throughout the study.

In case a study participant withdraws consent after having his data collected from him, the patient´s data will be anonymized.

15 Monitoring

External Monitoring is not planned.

The study is not powered to detect differences in clinical events. Events will be however collected and adjudicated by consensus of two cardiologists blinded to the allocation group as an additional procedure to monitor and guarantee the safety of the patients.

16 Use of radiation

There is no use of radiation for research purposes and outside the clinical routine. According to StrlSchV and RöV, or only according to the justifying indication of a competent physician following §80 StrlSchV and §23 RöV, no additional application is necessary. The patient exposed to radiation would receive the same radiation (type, duration, intensity) as if he or she was not a participant of the study. (Signed statement attached).

17 Statistical analysis

Statistical analysis will be performed with Medcalc (Mariakerk, Belgium) and SPSS.

Power Calculation

The study is powered for the primary efficacy endpoint IMR (balloon versus sham). In the OxAMI-PICSO study [13], PICSO reduced the IMR in patients with no reflow after ST-elevation myocardial infarction from 45 [22-51] to 25[19-36]. In unselected coronary artery disease patients, Mangiacapra et al[27] reported a decrease in IMR from 27±11 to 19±9 after intracoronary enalaprilat; Suda et al reported a decrease in IMR from ~28[21-35] to ~19[17-

23] (P<0.0001, median decrease ~35%) in response to fasudil[28]. Luo[29] reported baseline IMR of 33 units in patients with microvascular dysfunction (i.e. our expected population), with a baseline SD of 7.6 units. When assuming a baseline IMR of 33 and conservatively using the pooled baseline SD of IMR 13 units of both treatment groups from Mangiacapra, and assuming a similar effect as that observed in the papers by Mangiacapra and Suda, this will result in an effect size of ~0.69 (~9/13). With this effect size, a two-sided one-sample t-test and a power of 80%, 19 patients are needed. To allow for 25% missing values for patients with missing Pd, Tmn or Pv values either during sham or balloon inflation ("IMR not measureable"), up to 6 additional patients will be included in case of incomplete data collection.

Statistical analysis

The index of microvascular resistance will be calculated as mean transit time (Tmn) x (distal arterial minus coronary sinus venous blood pressure (Pd-Pv)). The primary analysis will be done for the IMR measurement. The primary hypothesis is H0: μExp = μSham, versus H1: μExp ≠ μSham, where μExp and μSham are the expected values of the IMR with experimental intervention and sham intervention respectively. The primary analysis will be performed as within-subject comparison of the primary parameter. Statistical tests and effect estimates will be calculated using a linear mixed regression model with a patient random intercept, condition and period as well as their interaction as fixed factors and age and sex as covariates. The condition by period interaction is expected to be insignificant because the balloon from the experimental intervention will produce a transient increase in coronary sinus pressure which will normalize upon deflation and thus not generate any carryover effect. The primary comparison will be performed at a two-sided significance level of $\alpha=0.05$. Descriptive statistics will also be provided, secondary endpoints will be analyzed using Wilcoxon tests. No interim analysis is planned for this small cross-over study. Primary and secondary analyses will be performed in the ITT (intention to treat) population comprising all randomized patients. Analyses will be repeated in the per protocol population as sensitivity analysis comprising all patients without an a priori defined protocol violation. Bias is minimized by randomized sequence allocation based on a randomization list. The randomization ratio of the treatment sequences will be 1:1 without any stratification factors. Randomization with permuted blocks will be applied. The randomization list will be kept in safe and confidential custody. Randomization will be used within this trial allowing investigators to randomize patients via the eCRF. Role specific access rights and the need to confirm all details necessary for stratified randomization are incorporated within the process and will reduce the risk of misuse and unintended randomizations

18 References

1. Knuuti J, Wijns W, Saraste A, Capodanno D, Barbato E, Funck-Brentano C, Prescott E, Storey RF, Deaton C, Cuisset T, Agewall S, Dickstein K, Edvardsen T, Escaned J, Gersh BJ, Svitil P, Gilard M, Hasdai D, Hatala R, Mahfoud F, Masip J, Muneretto C, Valgimigli M, Achenbach S, Bax JJ and Group ESCSD. 2019 ESC Guidelines for the diagnosis and management of chronic coronary syndromes. *Eur Heart J*. 2020;41:407-477.
2. Suzuki H. Different definition of microvascular angina. *Eur J Clin Invest*. 2015;45:1360-6.
3. Villano A, Lanza GA and Crea F. Microvascular angina: prevalence, pathophysiology and therapy. *J Cardiovasc Med (Hagerstown)*. 2018;19 Suppl 1:e36-e39.

4. Konigstein M, Giannini F and Banai S. The Reducer device in patients with angina pectoris: mechanisms, indications, and perspectives. *Eur Heart J*. 2018;39:925-933.
5. Verheye S, Jolicoeur EM, Behan MW, Pettersson T, Sainsbury P, Hill J, Vrolix M, Agostoni P, Engstrom T, Labinaz M, de Silva R, Schwartz M, Meyten N, Uren NG, Doucet S, Tanguay JF, Lindsay S, Henry TD, White CJ, Edelman ER and Banai S. Efficacy of a device to narrow the coronary sinus in refractory angina. *N Engl J Med*. 2015;372:519-27.
6. Konigstein M, Bazan S, Revivo M and Banai S. Coronary Sinus Reducer implantation improves symptoms, ischaemia and physical capacity in patients with refractory angina unsuitable for myocardial revascularisation: a single-centre experience. *EuroIntervention*. 2018;14:e452-e458.
7. Konigstein M, Meyten N, Verheye S, Schwartz M and Banai S. The Coronary Sinus Reducer improves angina and ischaemia by redistribution of blood from non-ischaemic to ischaemic myocardium. *EuroIntervention*. 2015;11:728.
8. Konigstein M, Shofti R, Schwartz M and Banai S. Coronary sinus reducer for the treatment of chronic refractory angina pectoris-results of the preclinical safety and feasibility study. *Catheter Cardiovasc Interv*. 2018;92:1274-1282.
9. Stoller M, Traupe T, Khattab AA, de Marchi SF, Steck H and Seiler C. Effects of coronary sinus occlusion on myocardial ischaemia in humans: role of coronary collateral function. *Heart*. 2013;99:548-55.
10. Ido A, Hasebe N, Matsuhashi H and Kikuchi K. Coronary sinus occlusion enhances coronary collateral flow and reduces subendocardial ischemia. *Am J Physiol Heart Circ Physiol*. 2001;280:H1361-7.
11. Giannini F, Baldetti L, Ielasi A, Ruparelia N, Ponticelli F, Latib A, Mitomo S, Esposito A, Palmisano A, Chieffo A and Colombo A. First Experience With the Coronary Sinus Reducer System for the Management of Refractory Angina in Patients Without Obstructive Coronary Artery Disease. *JACC Cardiovasc Interv*. 2017;10:1901-1903.
12. Jolicoeur EM, Banai S, Henry TD, Schwartz M, Doucet S, White CJ, Edelman E and Verheye S. A phase II, sham-controlled, double-blinded study testing the safety and efficacy of the coronary sinus reducer in patients with refractory angina: study protocol for a randomized controlled trial. *Trials*. 2013;14:46.
13. De Maria GL, Alkhalil M, Borlotti A, Wolfrum M, Gaughran L, Dall'Armellina E, Langrish JP, Lucking AJ, Choudhury RP, Kharbanda RK, Channon KM and Banning AP. Index of microcirculatory resistance-guided therapy with pressure-controlled intermittent coronary sinus occlusion improves coronary microvascular function and reduces infarct size in patients with ST-elevation myocardial infarction: the Oxford Acute Myocardial Infarction - Pressure-controlled Intermittent Coronary Sinus Occlusion study (OxAMI-PICSO study). *EuroIntervention*. 2018;14:e352-e359.
14. Lehmann EL. Nonparametrics : Statistical Methods Based on Ranks, Revised.: Pearson; 1998: pages 76-81.

19 Financing

The study will be financed by own means of the center of cardiology of the university hospital Mainz (=Sponsor) and means of the W3-Professorship of Translational myocardial and cardiovascular function. No third-party funds are planned.

20 Publication Policy

In accordance with the WMA Declaration of Helsinki - Ethical Principles for Medical Research Involving Human Subjects, researchers, authors, sponsors, editors and publishers all have ethical obligations with regard to the publication and dissemination of the results of

research. Researchers have a duty to make publicly available the results of their research on human subjects and are accountable for the completeness and accuracy of their reports. All parties should adhere to accepted guidelines for ethical reporting. Negative and inconclusive as well as positive results must be published or otherwise made publicly available. Sources of funding, institutional affiliations and conflicts of interest must be declared in the publication. Reports of research not in accordance with the principles of this Declaration should not be accepted for publication.

The PI of this study, recognizing the seminal importance of this investigation, is committed to the unrestricted and widespread dissemination of all primary and secondary endpoint results and tertiary analyses. At the conclusion of the study, a abstract reporting the primary results will be prepared by the Principal Investigators and presented at an annual scientific meeting (e.g., Transcatheter Cardiovascular Therapeutics, EuroPCR, the American Heart Association, the American College of Cardiology, or the European Society of Cardiology meeting). A publication will similarly be prepared for publication in a reputable scientific journal.

Following analysis and presentation of the primary endpoint results, active participation of all study group members, will be solicited for data analysis and abstract and manuscript preparation and therefore included as co-authors. Submission of all abstracts and publications regarding the primary endpoint and secondary endpoints from the study requires approval by the Principal Investigators after review by all members of the study group.

Mainz, 14.09.2020
