# Supplementary material for: Coronary Venous Pressure and Microvascular Hemodynamics in Patients With Microvascular Angina: A Randomized Clinical Trial
Source: JAMA Cardiol. 2023 Aug 23;8(10):979–83. doi: 10.1001/jamacardio.2023.2566 (PMC10448373; doi:10.1001/jamacardio.2023.2566)
Supplement: Supplement 2. — eTable. Patient Characteristics eMethods. eFigure 1. Impact of Balloon Expansion on Coronary Flow Capacity eFigure 2. Correlation Between the Decrease in IMR Following Balloon Inflation and Sham (Resting) IMR eFigure 3. Proposed Mechanism eReferences. [file jamacardiol-e232566-s002.pdf]

Supplementary Online Content

Ullrich H, Hammer P, Olschewski M, Münzel T, Escaned J, Gori T. Coronary venous pressure and microvascular hemodynamics in patients with microvascular angina: a randomized clinical trial. *JAMA Cardiol*. Published online August 23, 2023. doi:10.1001/jamacardio.2023.2566

eTable. Patient Characteristics

eMethods.

eFigure 1. Impact of Balloon Expansion on Coronary Flow Capacity

eFigure 2. Correlation Between the Decrease in IMR Following Balloon Inflation and Sham (Resting) IMR

eFigure 3. Proposed Mechanism

eReferences

This supplementary material has been provided by the authors to give readers additional information about their work.

© 2023 Ullrich H et al. *JAMA Cardiology.*

eTable. Patient Characteristics

	N
**Women**	11
**Age (years)**	69[64-75]
**Diabetes**	2
**Hypercholesterolemia**	6
**Hypertension**	15
**Smoking**	3
**Prior percutaneous intervention**	2 (RCA, LCX)
**Inflammatory diseases**	2 (psoriasis, rheumatoid arthritis)
**Hyperemic agent used**	
Intracoronary papaverine, 14mg bolus	1
Intravenous adenosine, 140micrgr/kg/minute	19

eMethods

a. Hemodynamic assessments

In the current study, we provide evidence that an (acute) increase in coronary sinus pressure leads to a decrease in Tmn (inverse of flow), FFR and IMR. Of note, while remaining above the threshold accepted for intervention in this group of patients without epicardial disease, FFR decreased during balloon inflation (P=0.003). This is compatible with the larger degree of hyperemia (as confirmed by the lower Tmn) reached during balloon inflation: since the gradient across a stenosis (or diffuse atherosclerosis, as in this case) is proportional to the square of blood flow velocity, a decrease in microvascular resistance would be expected to be associated with a drop in FFR. As well, the (numerical) increase in coronary flow reserve and the proportionally larger change in hyperemic (as compared to resting) resistances suggests that the increase in coronary sinus pressure did not reset flow to produce a “semi-hyperemic” state (i.e., a parallel decrease in resting and hyperemic resistances), but it rather improved the capacity of the blood/microcirculation system to react to vasodilator stimuli.

Coronary flow capacity (CFC) was also displayed to identify the impact of balloon inflation on this parameter that was designed to describe hemodynamics in a more comprehensive way as compared to coronary flow reserve alone ([1,2]). The concept of CFC was proposed by Johnson and Gould to provide a categorical assessment of coronary hemodynamics resulting from a combination of hyperemic coronary flow (inverse of Tmn) and coronary flow reserve (CFR). CFR is a well-validated index that is influenced by both epicardial and microcirculatory function and provides information on long-term adverse events (for instance, [3]). The limitation of CFR is that, being a ratio of hyperemic to resting blood flow, it is influenced by both parameters and is unable to distinguish in changes in either one. CFC was proposed to overcome this issue[4]. The concept of CFC is based on a categorical assessment of

© 2023 Ullrich H et al. *JAMA Cardiology.*

the combination of hyperemic myocardial blood flow and CFR. Importantly, CFC was found to offer superior prognostic efficacy for long-term clinical outcomes as compared to CFR alone[2]. A normal CFC, indicating no myocardial ischemia is described as CFR ≥2.80 and 1/Tmn ≥3.70; mildly reduced CFC is with CFR 2.80-2.10 and 1/Tmn 3.70-2.56; moderately reduced CFC is given by a combination of CFR 2.10-1.70 with 1/Tmn 2.56-2.00; and severely reduced CFC is CFR <1.70, 1/Tmn <2.00. In our study, coronary flow capacity improved in response to balloon occlusion from an intermediate ischemia to a non-ischemic region.

b. Clinical implications

There is a number of potential clinical implications of these findings. A temporary occlusion of the coronary sinus has been tested in the setting of myocardial infarction using the PICSO device. Studies using this device (currently not available for use) have indeed shown acute improvements in the degree of microvascular obstruction, microvascular resistance, and area of infarction following STEMI of the anterior wall[5-8]. In terms of chronic therapy, the coronary sinus reducer device has emerged as a valuable tool for the management of refractory angina, as confirmed by the COSIRA trial, a randomized, patient-blinded, sham-controlled study which demonstrated an improvement in Canadian Cardiovascular Society angina but also in exercise tolerance in patients who received the reducer[9]. Typically, patients with obstructive coronary disease not amenable to revascularization (e.g. patients with previous by-pass grafts, diffuse disease etc.) are treated with this device, and a number of large registries have essentially reproduced the results of the COSIRA trial[10-15].

Beyond the treatment of patients with obstructive epicardial disease, some evidence from isolated case reports or small case series is available suggesting that the reducer may improve myocardial perfusion and decrease symptoms in patients with microvascular angina[16,17]. While the current mechanistic study provides the evidence that acute changes in CS pressure may reduce microvascular resistance, it was not designed to address whether: 1. this effect can be maintained over prolonged period of times (for instance, after implantation of a coronary sinus reducer) and 2. whether this leads to sustained improvements in angina and myocardial perfusion in patients with microvascular disease.

With regards to the first question, at least three studies are currently recruiting: the study by Dr De Silva (NCT04606459, NCT05492110) is a randomised double-blinded sham-controlled will recruit 54 patients to investigate the reducer´s effect on myocardial perfusion using MRI. A nested mechanistic substudy will also test the effect of the CS Reducer on measures of coronary microcirculatory physiology. The study by Professor Lerman has enrolled 30 patients with microvascular angina to test the impact of the coronary sinus reducer on invasively assessed coronary flow reserve and microvascular resistances at 4 months (NCT04523168). In the COSIRA-II trial (NCT05102019) has a single-arm substudy enrolling patients without epicardial disease. Finally, with regards to the seconds question, the COSIMA trial (NCT04606459) is a randomized, multicentric trial enrolling 144 patients with microvascular angina who are randomized to reducer or optimal medical therapy. Endpoint of this trial is CCS angina, with a number of parameters of exercise capacity and quality of life as secondary endpoints. Of note this list is probably incomplete as an increasing number of groups engage in this field of research.

© 2023 Ullrich H et al. *JAMA Cardiology.*

While it is not designed to address the two questions above, the current study provides the first evidence of a role of the venous circulation in coronary hemodynamics and represents the mechanistic background for these studies.

© 2023 Ullrich H et al. *JAMA Cardiology.*

eFigure 1. Impact of balloon expansion on coronary flow capacity

The solid circle represents CFC at baseline indicating moderate ischaemia region. The empty circle (dotted lines) indicates CFC during CS occlusion (no ischemia region)

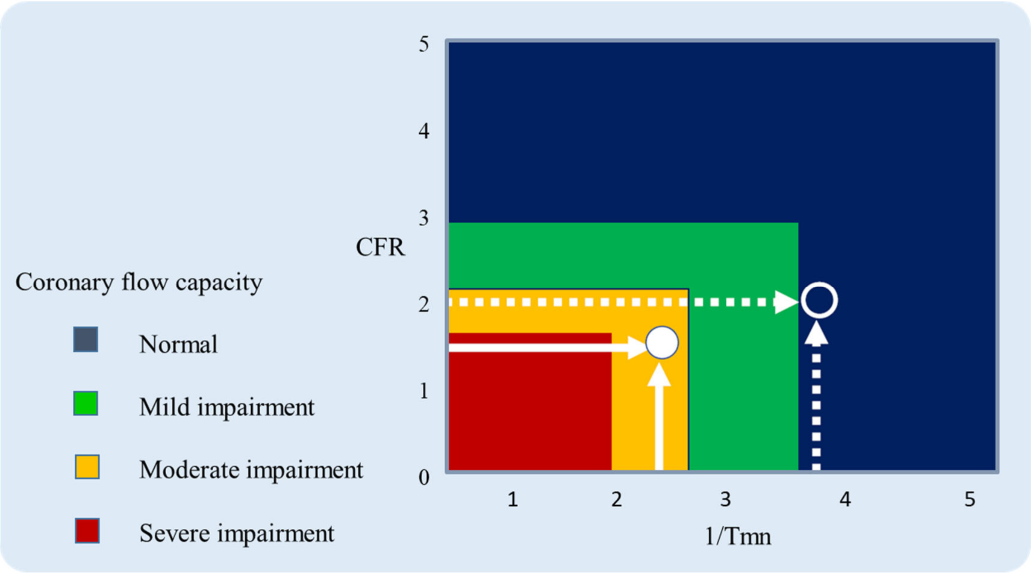

eFigure 2. Correlation between the decrease in IMR following balloon inflation and sham (resting) IMR

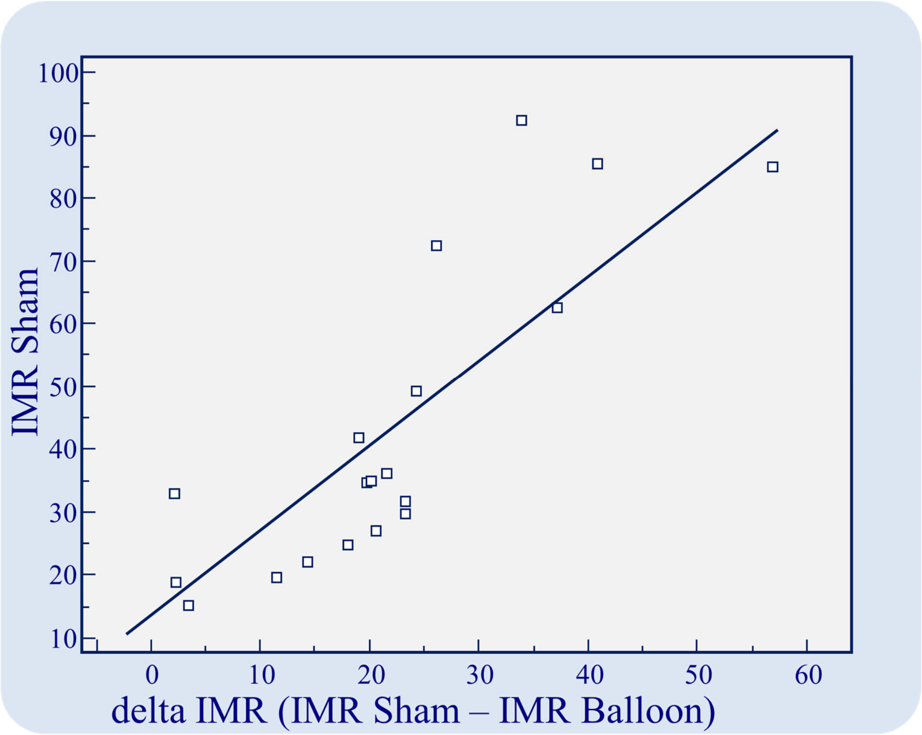

© 2023 Ullrich H et al. *JAMA Cardiology.*

eFigure 3. proposed mechanism

An increase in retrograde pressure would cause an increase in total myocardial blood volume though recruitment of capillaries. This would additionally be associated with a decrease in viscosity, resulting in decreased resistances (more pronounced during hyperemia).

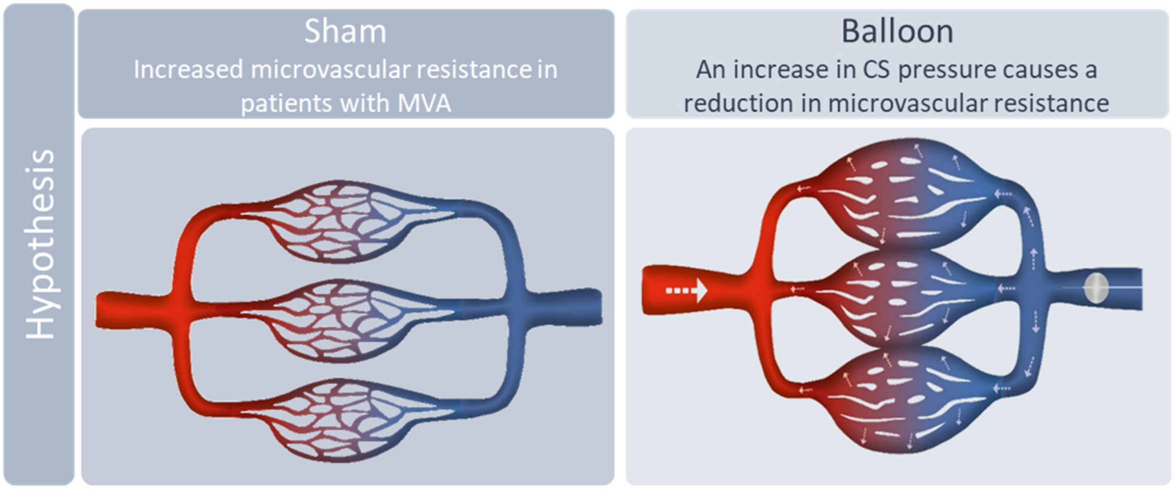

eReferences

1. Hamaya R, Yonetsu T, Kanaji Y, et al. Diagnostic and Prognostic Efficacy of Coronary Flow Capacity Obtained Using Pressure-Temperature Sensor-Tipped Wire-Derived Physiological Indices. *JACC Cardiovasc Interv* 2018; **11**(8): 728-37.
2. van de Hoef TP, Echavarria-Pinto M, van Lavieren MA, et al. Diagnostic and Prognostic Implications of Coronary Flow Capacity: A Comprehensive Cross-Modality Physiological Concept in Ischemic Heart Disease. *JACC Cardiovasc Interv* 2015; **8**(13): 1670-80.
3. Chamuleau SA, van Eck-Smit BL, Meuwissen M, et al. Long-term prognostic value of CFVR and FFR versus perfusion scintigraphy in patients with multivessel disease. *Neth Heart J* 2007; **15**(11): 369-74.
4. Johnson NP, Gould KL. Integrating noninvasive absolute flow, coronary flow reserve, and ischemic thresholds into a comprehensive map of physiological severity. *JACC Cardiovasc Imaging* 2012; **5**(4): 430-40.
5. De Maria GL, Alkhalil M, Borlotti A, et al. Index of microcirculatory resistance-guided therapy with pressure-controlled intermittent coronary sinus occlusion improves coronary microvascular function and reduces infarct size in patients with ST-elevation myocardial infarction: the Oxford Acute Myocardial Infarction - Pressure-controlled Intermittent Coronary Sinus Occlusion study (OxAMI-PICSO study). *EuroIntervention* 2018; **14**(3): e352-e9.
6. Egred M, Bagnall A, Spyridopoulos I, et al. Effect of Pressure-controlled intermittent Coronary Sinus Occlusion (PiCSO) on infarct size in anterior STEMI: PiCSO in ACS study. *Int J Cardiol Heart Vasc* 2020; **28**: 100526.
7. Scarsini R, Terentes-Printzios D, Shanmuganathan M, et al. Pressure-controlled intermittent coronary sinus occlusion improves the vasodilatory microvascular capacity and reduces myocardial injury in patients with STEMI. *Catheter Cardiovasc Interv* 2022; **99**(2): 329-39.

© 2023 Ullrich H et al. *JAMA Cardiology.*

8. van de Hoef TP, Nijveldt R, van der Ent M, et al. Pressure-controlled intermittent coronary sinus occlusion (PICSO) in acute ST-segment elevation myocardial infarction: results of the Prepare RAMSES safety and feasibility study. *EuroIntervention* 2015; **11**(1): 37-44.
9. Verheye S, Jolicoeur EM, Behan MW, et al. Efficacy of a device to narrow the coronary sinus in refractory angina. *N Engl J Med* 2015; **372**(6): 519-27.
10. Abawi M, Nijhoff F, Stella PR, et al. Safety and efficacy of a device to narrow the coronary sinus for the treatment of refractory angina: A single-centre real-world experience. *Neth Heart J* 2016; **24**(9): 544-51.
11. Giannini F, Baldetti L, Konigstein M, et al. Safety and efficacy of the reducer: A multi-center clinical registry - REDUCE study. *Int J Cardiol* 2018; **269**: 40-4.
12. Konigstein M, Ponticelli F, Zivelonghi C, et al. Long-term outcomes of patients undergoing coronary sinus reducer implantation - A multicenter study. *Clin Cardiol* 2021; **44**(3): 424-8.
13. Ponticelli F, Khokhar AA, Leenders G, et al. Safety and efficacy of coronary sinus narrowing in chronic refractory angina: Insights from the RESOURCE study. *Int J Cardiol* 2021; **337**: 29-37.
14. Verheye S, Agostoni P, Giannini F, et al. Coronary sinus narrowing for the treatment of refractory angina: a multicentre prospective open-label clinical study (the REDUCER-I study). *EuroIntervention* 2021; **17**(7): 561-8.
15. Wlodarczak S, Rola P, Jastrzebski A, et al. Coronary Sinus Reducer implantation in refractory angina. Short-term outcome of Lower Silesia Sinus Reducer Registry (LSSRR). *Kardiol Pol* 2023.
16. Giannini F, Baldetti L, Ielasi A, et al. First Experience With the Coronary Sinus Reducer System for the Management of Refractory Angina in Patients Without Obstructive Coronary Artery Disease. *JACC Cardiovasc Interv* 2017; **10**(18): 1901-3.
17. Cheng K, Keramida G, Baksi AJ, de Silva R. Implantation of the coronary sinus reducer for refractory angina due to coronary microvascular dysfunction in the context of apical hypertrophic cardiomyopathy-a case report. *Eur Heart J Case Rep* 2022; **6**(11): ytac440.

© 2023 Ullrich H et al. *JAMA Cardiology.*
