# Supplementary material for: Coronary Venous Pressure and Microvascular Hemodynamics in Patients With Microvascular Angina: A Randomized Clinical Trial
Source: JAMA Cardiol. 2023 Aug 23;8(10):979–83. doi: 10.1001/jamacardio.2023.2566 (PMC10448373; doi:10.1001/jamacardio.2023.2566)
Supplement: Supplement 3. — Data Sharing Statement [file jamacardiol-e232566-s003.pdf]

Data Sharing Statement

Ullrich H, Hammer P, Olschewski M, Münzel T, Escaned J, Gori T. Coronary venous pressure and microvascular hemodynamics in patients with microvascular angina: a randomized clinical trial. *JAMA Cardiol.* Published online August 23, 2023. doi:10.1001/jamacardio.2023.2566

Data available:
Yes
Data types:
Deidentified participant data
How to access data:
tommaso.gori@unimedizin-mainz.de
When available:
With publication

Document types:
None
Who can access the data:
Upon motivated legitimate request.
Types of analyses:
No a priori limitations
Mechanisms of data availability:
after approval of a proposal and upon signature of a DSA.
Any additional restrictions: None
